# Supplementary material for: Kaptive 2.0: updated capsule and lipopolysaccharide locus typing for the Klebsiella pneumoniae species complex
Source: Microb Genom. 2022 Mar 21;8(3):000800. doi: 10.1099/mgen.0.000800 (PMC9176290; doi:10.1099/mgen.0.000800)
Supplement: Supplementary material 1 [file mgen-8-0800-s001.pdf]

Supplementary Table 1. Novel K loci identified in this study

K locus	Reference genome/read accession	Length (bp)	#CDS	#genomes
KL171	GCF_001874715.1	24,175	21	1
KL172	ERR3449083	27,593	23	1
KL173*	GCF_003990375.1	25,719	21	4
KL174	ERR315145	24,175	20	15
KL175	ERR4367681	28,533	25	1
KL176*	ERR3448903	25,166	19	5
KL177	T7-221	29,929	25	8
KL178	T7-392	28,865	24	3
KL179	GCF_900407305.1	29,218	23	2
KL180	T7-177	30,215	24	7
KL181*	ERR4367597	27,966	23	9
KL182	JAJHNT000000000	26,104	22	2
KL183	T7-391	22,626	19	33
KL184*	ERR4367641	26,378	24	2
KL185*	GCF_900493845.1	26,462	21	3
KL186	GCF_002247665.1	25,893	21	5

*K loci where IS were manually removed from the reference sequence
